# Supplementary material for: Antimicrobial Resistance Risk Assessment of Vibrio parahaemolyticus Isolated from Farmed Green Mussels in Singapore
Source: Microorganisms. 2023 Jun 5;11(6):1498. doi: 10.3390/microorganisms11061498 (PMC10301002; doi:10.3390/microorganisms11061498)
Supplement: Supplementary file 1 [file microorganisms-11-01498-s001.zip › microorganisms-2376101-supplementary.pdf]

Table S1 – Confirmation of antimicrobial resistance of *Vibrio parahemolyticus* bacterial isolates via disc diffusion method from green mussels.

	Treatment		
	Ampicillin	Penicillin G	Tetracycline
Bacterial isolates counts	98	105	94
Resistant disc diffusion results (%)	97 (98.9)	105 (100)	92 (97.8)

Table S2 – Antimicrobial susceptibility profile of *V. parahaemolyticus* isolates via disc diffusion method isolated from green mussels.

Antibiotics	Intepretive Results (Farm Module)			Intepretive Results (Retail Module)		
	Sensitive (%)	Intermediate (%)	Resistant (%)	Sensitive(%)	Intermediate (%)	Resistant (%)
Ampicillin (10 µg)	2 (3.1)	17 (26.1)	46 (70.8)	9 (7.80)	13 (11.2)	94 (81.0)
Ampicillin/ sulbactam (20 µg)	65 (100)	-	-	100 (86.2)	2 (1.70)	14 (12.1)
Pencillin G (10 unit)	-	-	65 (100)	4 (3.40)	-	112 (96.6)
Tetracycline (30 µg)	60 (92.3)	2 (3.10)	3 (4.60)	95 (81.9)	5 (4.30)	16 (13.8)
Cefotaxime (30 µg)	58 (89.2)	7 (10.8)	-	105 (90.5)	11 (9.50)	-
Ciprofloxacin (5 µg)	63 (96.9)	2 (3.08)	-	100 (86.2)	16 (13.8)	-
Sulfamethoxazole/ Trimethoprim (25 µg)	65 (100)	-	-	116 (100)	-	-
Chloramphenicol (30 µg)	65 (100)	-	-	116 (100)	-	-

Table S3 – Standard errors of occurrence and concentration data of haemolytic *V. parahaemolyticus* within the farm-to-home and retail-to-home chain scenarios in green mussels.

			Farm to home				Retail to home			
			Unsupplemented (SE)	Ampicillin (SE)	Penicillin G (SE)	Tetracycline (SE)	Unsupplemented (SE)	Ampicillin (SE)	Penicillin G (SE)	Tetracycline (SE)
	Prevalence (Farm/Retail)		6.02E-05	5.45E-05	4.95E-05	3.48E-05	2.26E-05	4.21E-05	3.74E-05	3.47E-05
Concentration (LogCFU/g)	Farm	Pre-harvest	4.05E-04	4.36E-04	3.91E-04	2.53E-04	N.A.	N.A.	N.A.	N.A.
		Post-harvest	4.24E-04	5.17E-04	4.55E-04	3.43E-04	N.A.	N.A.	N.A.	N.A.
	Retail	Retail-start	4.27E-04	5.21E-04	4.54E-04	3.45E-04	6.06E-04	4.10E-04	5.18E-04	3.30E-04
		Retail-end	4.26E -04)	5.21E-04	4.54E-04	3.45E-04	6.05E-04	4.09E-04	5.18E-04	3.30E-04
	Home	Home	4.24E-04	5.17E-04	4.52E-04	3.43E-04	5.98E-04	4.18E-04	5.22E-04	3.22E-04
	Cooking	Average	7.15E-04	6.14E-04	6.21E-04	3.98E-04	6.73E-04	4.95E-04	4.92E-04	2.07E-04
		Minimally cooked	7.68E-04	6.69E-04	6.32E-04	4.91E-04	7.47E-04	5.33E-04	6.65E-04	5.04E-04
		Moderately cooked	6.80E-04	8.46E-04	7.11E-04	5.75E-04	8.96E-04	5.61E-04	6.85E-04	2.06E-04
		Highly cooked	1.86E-04	1.18E-04	1.51E-04	4.20E-06	4.30E-04	2.38E-04	9.59E-05	0

Table S4 – Standard errors of risk estimates data across all scenarios in green mussels.

		Farm to home			Retail to home		
		$P_{ill,serving}$ (SE)	$P_{ill,yearly}$ (SE)	N_{cases} (SE)	$P_{ill,serving}$ (SE)	$P_{ill,yearly}$ (SE)	N_{cases} (SE)
Unsupplemented	Average	3.93E-05	1.28E-04	6.12E-01	6.07E-05	1.56E-04	7.45E-01
	Minimally cooked	1.92E-04	3.20E-04	1.53	2.32E-04	2.77E-04	1.33
	Moderately cooked	2.09E-05	2.81E-04	1.34	7.33E-05	2.31E-04	1.10
	Highly cooked	1.03E-07	2.45E-05	1.17E-01	1.37E-06	1.13E-04	5.43E-01
Ampicillin	Average	2.41E-05	1.40E-04	6.72E-01	3.54E-05	1.15E-04	5.52E-01
	Minimally cooked	1.23E-04	2.10E-04	1.01	1.09E-04	2.84E-04	1.36
	Moderately cooked	1.31E-05	2.66E-04	1.27	2.95E-05	2.10E-04	1.01
	Highly cooked	3.63E-08	1.42E-05	6.79E-02	1.70E-07	3.80E-05	1.82E-01
Penicillin G	Average	2.74E-05	9.71E-05	4.65E-01	2.92E-05	1.14E-04	5.44E-01
	Minimally cooked	8.19E-05	2.26E-04	1.08	1.42E-04	1.89E-04	9.07E-01
	Moderately cooked	1.55E-05	2.02E-04	9.65E-01	1.28E-05	1.97E-04	9.43E-01
	Highly cooked	7.38E-08	2.11E-05	1.01E-01	4.38E-08	1.55E-05	7.41E-02
Tetracycline	Average	6.35E-06	8.81E-05	4.22E-01	1.10E-06	3.88E-05	1.86E-01
	Minimally cooked	3.47E-05	2.44E-04	1.17	5.78E-06	2.40E-04	1.15
	Moderately cooked	1.18E-06	1.23E-04	5.88E-01	7.91E-08	3.05E-05	1.46E-01
	Highly cooked	8.14E-10	5.74E-07	2.75E-03	0	0	0
